# The double burden of disease among mining workers in Papua, Indonesia: at the crossroads between Old and New health paradigms

**DOI:** 10.1186/s12889-016-3630-8

**Published:** 2016-09-08

**Authors:** Rodrigo Rodriguez-Fernandez, Nawi Ng, Dwidjo Susilo, John Prawira, Michael J. Bangs, Rachel M. Amiya

**Affiliations:** 1 Non-Communicable Diseases, International SOS, London, UK; 2 Department of Public Health and Clinical Medicine, Epidemiology and Global Health, Umeå University, Umeå, Sweden; 3 Center for Health Policy and Management, Faculty of Medicine, Gadjah Mada University, Yogyakarta, Indonesia; 4 Freeport Public Health and Malaria Control, International SOS, Papua, Indonesia; 5 Department of Family Nursing, Graduate School of Medicine, University of Tokyo, Tokyo, Japan; 6 NCD Asia Pacific Alliance, Tokyo, Japan

**Keywords:** Non-communicable disease, Infectious disease, Comorbidity, Data visualization, Primary health care, Indonesia

## Abstract

**Background:**

As the global shift toward non-communicable diseases overlaps with the unfinished agenda of confronting infectious diseases in low- and middle-income countries, epidemiological links across both burdens must be recognized. This study examined the non-communicable disease-infectious disease overlap in the specific comorbidity rates for key diseases in an occupational cohort in Papua, Indonesia.

**Methods:**

Diagnosed cases of ischaemic heart disease, stroke, hypertension, diabetes (types 1 and 2), chronic obstructive pulmonary disease, asthma, cancer, HIV and AIDS, tuberculosis, and malaria were extracted from 22,550 patient records (21,513 men, 1037 women) stored in identical electronic health information systems from two clinic sites in Papua, Indonesia. Data were collected as International Classification of Diseases, 10th Revision, entries from records spanning January-December 2013. A novel application of *Circos* software was used to visualize the interconnectedness between the disease burdens as overlapping prevalence estimates representing comorbidities.

**Results:**

Overall, NCDs represented 38 % of all disease cases, primarily in the form of type 2 diabetes (*n* = 1440) and hypertension (*n* = 1398). Malaria cases represented the largest single portion of the disease burden with 5310 recorded cases, followed by type 2 diabetes with 1400 cases. Tuberculosis occurred most frequently alongside malaria (29 %), followed by chronic obstructive pulmonary disease (19 %), asthma (17 %), and stroke (12 %). Hypertension-tuberculosis (4 %), tuberculosis-cancer (4 %), and asthma-tuberculosis (2 %) comorbidities were also observed.

**Conclusions:**

The high prevalence of multimorbidity, preponderance of non-communicable diseases, and extensive interweaving of non-communicable and infectious disease comorbidities highlighted in this cohort of mining workers in Papua, Indonesia reflect the markedly double disease burden increasingly plaguing Indonesia and other similar low- and middle-income countries – a challenge with which their over-stretched, under-resourced health systems are ill-equipped to cope. Integrated, person-centered treatment and control strategies rooted in the primary healthcare sector will be critical to reverse this trend.

## Background

On top of the unfinished agenda of combating infectious diseases (IDs) in low- and middle-income countries (LMICs), development, industrialization, urbanization, and an aging population are driving a rising epidemic of non-communicable diseases (NCDs). NCDs such as heart disease, stroke, diabetes mellitus, cancer, asthma, and chronic respiratory diseases now account for approximately 60 % of total mortality worldwide, with roughly 80 % of the chronic NCD deaths occurring in LMICs [1]. In particular, the South-East Asia region bears a disproportionate portion of global disease overall [2] and has faced a steadily rising NCD burden [3]. Countries like Indonesia are thus heavily hit with a *double* burden of concurrent infectious and non-communicable afflictions.

Indonesia’s increasing NCD burden is in line with the growth of key metabolic risk factors including hypertension, high blood glucose, and obesity – primarily the influence of expanding lifestyle-related risk behaviours such as poor diet, physical inactivity, and smoking. This shifting disease burden is reflected in the ten leading causes of Indonesia’s disability-adjusted life years, ranked in 2010 as stroke, tuberculosis (TB), traffic accidents, diarrhoea, cardiac ischemia, diabetes, lower back pain, depression, respiratory infections, and neonatal encephalopathy [4]. Notably, NCDs occupy half of this list, including the uppermost slot, while TB and diarrheal diseases also persist as prominent causes of disability and death. This intersection of old and new disease paradigms has translated to an increased strain on the country’s existing health care system, which has had to expand its scope of services in order to deal with the increasing number of NCD cases while at the same time coping with the ongoing burdens of TB, malaria, and HIV and AIDS.

Adding to the unprecedented task of addressing the emerging double disease burden, Indonesia’s Ministry of Health has recently introduced a universal healthcare scheme that will endeavour to provide healthcare access for over 180 million people distributed across its vast, variously populated archipelago, with plans to expand coverage to all remaining citizens by 2019 [5]. Significant complexity has also been created by Indonesia’s decentralization policy beginning in 2000, with the responsibility for budgeting and development planning largely devolved to more than 539 autonomous regencies [6]. Moreover, Indonesian health expenditure as a percentage of GDP, approximately 2.2 %, is below most of its South-East Asian neighbours, which also score better on most other health indicators [7]. It is therefore imperative to better define Indonesia’s current epidemiological panorama regarding area-specific emerging disease patterns so as to effectively allocate resources amongst its diverse localities.

Amongst Indonesia’s current 34 provinces, health indicators in the two provinces on the western side of the island of New Guinea (Papua and West Papua, hereafter collectively referred to as ‘Papua’) remain the poorest in Indonesia, wherein difficulties of access and availability of health services result in a high proportion of the population being under-served. Similar to patterns seen in other LMICs, the rapid industrialization in Papua has brought with it an increase in NCDs within a very short time frame. Not only does Papua face the harshest ID burden within Indonesia (and arguably most of Asia), but it is now saddled with the emergence of NCD risk factors and an associated NCD prevalence markedly above national averages [8].

Numerous commentaries have highlighted the emerging, concurrent burden of non-communicable and infectious disease in LMICs, and the consequent negative social, economic, and political effects [9–12]. However, an underexplored question to be answered before setting priorities and allocating (or reallocating) resources for health beyond 2015 is whether the sharp demarcation between IDs and NCDs, which is apparent in most projections of global mortality and disease burden, is justified when both may impact the same individuals and societies with near equivalence. Some efforts to challenge this false dichotomy that has emerged between NCDs and IDs in LMICs have been made [9]; however, data that describe the potential interconnectedness between specific NCD and ID burdens is scant. Hence, this epidemiological study aims to describe, for the first time, the prevalence and distribution of overlapping NCDs and IDs within a selected population in Papua, Indonesia over a 12-month period by using medical surveillance data from primary and secondary health facilities.

## Methods

### Study setting and participants

This research forms part of the ongoing Cardiovascular Outcomes in a Papuan Population and Estimation of Risk (COPPER) Study, set in a southern area of Papua Province, Indonesia. The study population comprised surface and underground mine workers aged 18 to 68 years who were employed by a multinational company and participated in annual health examinations performed at two mine-related health facilities from 1 January 2013 through 31 December 2013. One site (Kuala Kencana) is located approximately 20 km from the district town of Timika and the second (Tembagapura) within an isolated mountainous area in the Papuan highlands, approximately 2000 m above sea level. At the time of this study, the mining company employed approximately 22,558 people, of whom 71 % worked at the highlands site (up to 4200 m above sea level) and 29 % in the lowlands; 94 % were male, with 70 % below the age of 45 years.

### Data collection and analysis

The 1-year prevalences of ischaemic heart disease (IHD), stroke, hypertension, diabetes (types 1 and 2), chronic obstructive pulmonary disease (COPD), asthma, cancer, HIV and AIDS, TB, and malaria were extracted from patient records stored in the electronic health information systems of the two clinics for the period spanning 1 January-31 December 2013. Patient data are routinely collected as part of standard clinical and public health practice and stored in MS Access (Microsoft Corp, Redmond, WA, USA). Data were available for 21,513 men and 1037 women (mean age: 44.0 ± 10.7 years).

Data were collected in the form of International Classification of Diseases (ICD)-10 entries. In cases where multiple entries were present for a single patient, data were pooled and all entries screened concurrently for that patient. Extracted entries were filtered for the diseases of interest, wherein comorbidities were identified from any two of the relevant ICD-10 diagnostic codes in a patient’s record. The proportion of comorbidity was calculated for each disease as the percentage of total cases occurring conjointly with each of the other ten diseases of interest. Patients that presented more than once for the same disease within the 12-month period were recorded only once for that disease.

### Data visualization: *Circos*

The *Circos* software package [13] was then used to visualize the comorbidity relationships among sampled diseases as well as the individual burden of disease represented by total number of cases for each disease. The resulting visualization takes the form of a circular weighted graph, what we have termed a *multimorbidity wheel*. Each disease is represented by a separate colour. Given the small number of retrieved cases for both HIV and AIDS, both where group together. The outer periphery of the wheel depicts the absolute number of cases registered for each disease within the 12-month period. For each disease, the length of the outer arc represents the total sum of cases. Cases within a disease that also presented with a second disease are visualized as an internal ribbon, linking the two. The size (width) of the ribbon represents the relative frequency of this relationship; i.e., apparent relationships that are more frequent are more visually obvious [13].

Kryzwinski et al. [13] originally introduced *Circos* as a data visualization software showing the chromosomal relationships between various biological species. However, the tool is also an effective means of representing other data, with the circular layout ideal for exploring relationships between various entities. Herein, we use the software to show the interconnectedness between different disease burdens present in the study sample.

### Ethics statement

This study utilized secondary data collected as part of routine operations of the sampled health facilities. Thus, it was difficult to obtain informed consent retrospectively from the employees concerned. As only anonymous data are used herein, it was judged unnecessary to collect such consent for the purposes of this study. All data collected were properly secured and accessible only to the authors conducting the analyses. Study protocols were reviewed and approved by the Science Review Committee of International SOS.

## Results

Women (*n* = 812) represented 3.6 % of the total sample. Mean age was 39.5 ± 8.1 years for men and 36.8 ± 7.6 years for women. Papuan employees made up roughly one-third of the study population, while non-Papuan Indonesians and foreign nationals represented 62.4 and 3.3 %, respectively.

**Fig. 1:**
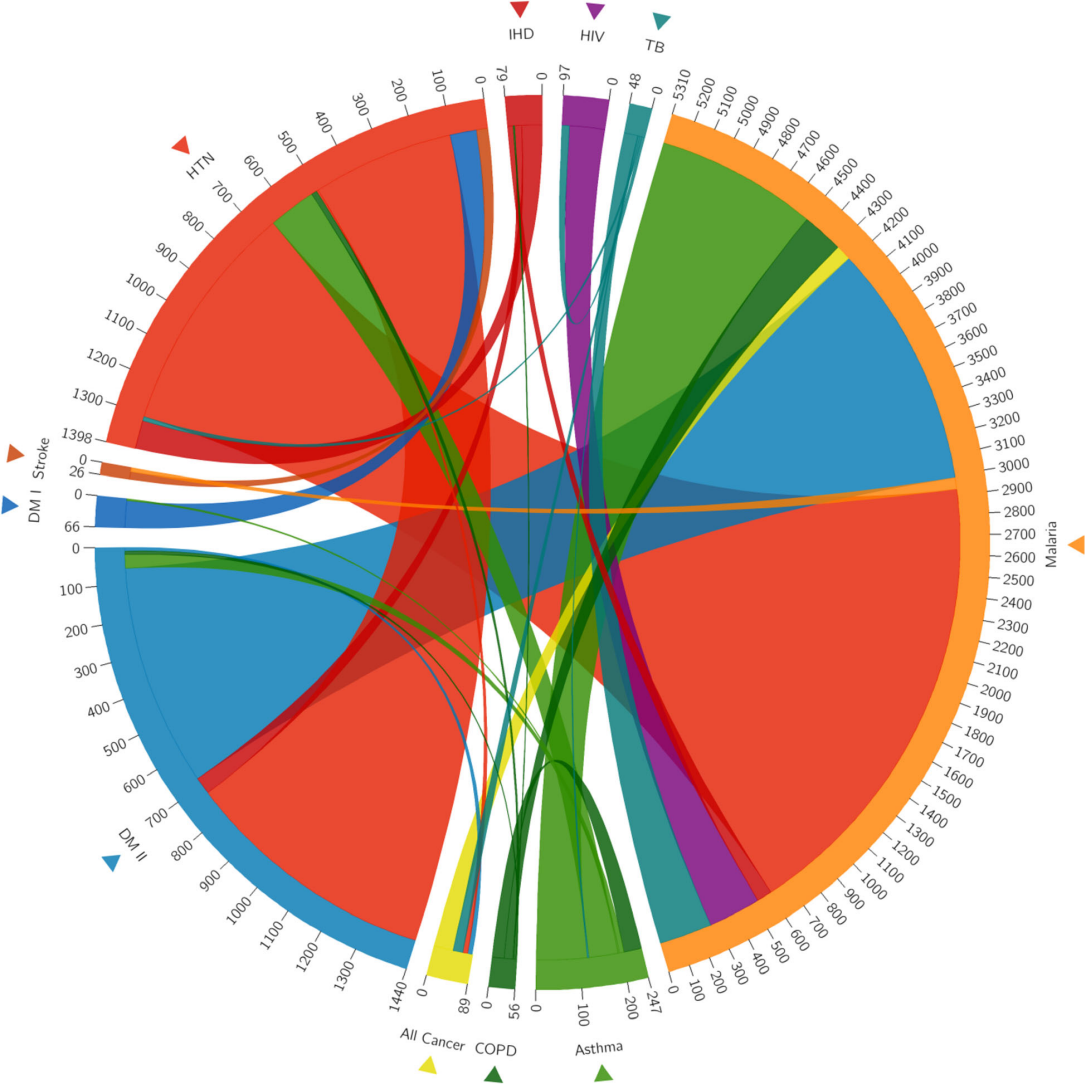
Multimorbidity wheel showing relationships between non-communicable and infectious disease burdens

Table 1 presents the recorded comorbidity proportions of 8856 outpatient and inpatient cases between the 11 diseases, while Fig. 1 visualizes the overlapping disease burdens using a *Circos*-generated multimorbidity wheel. The most common comorbidity was malaria, with 29.2 % of all such cases also having TB. This high comorbidity rate was followed closely by hypertension cases also having presented with an episode of stroke during the study period – 23.1 %. The proportion of hypertensive cases presenting with type 1 diabetes was 21.2 %, and the proportion of malaria cases with COPD was 19.6 %.

**Table 1:** Comorbidity proportions between defined non-communicable and infectious diseases

	Disease II											
Disease I		IHD	HTN	Stroke	DM I	DM II	All Cancer	COPD	Asthma	Malaria	TB	HIV and AIDS
	IHD		-	-	-	-	-	1.79 %	-	-	-	-
	HTN	19 %		23.08 %	21.21 %	-	1.12 %	5.36 %	10.12 %	-	4.17 %	-
	Stroke	-	-		-	-	-	-	-	-	-	-
	DM I	-	-	-		-	-	-	0.40 %	-	-	-
	DM II	6.33 %	5.36 %	-	0.00 %		1.12 %	1.79 %	1.62 %	-	-	-
	All Cancer	-	-	-	-	-		-	-	-	-	-
	COPD	1.79 %	-	-	-	-	-		6.07 %	-	-	-
	Asthma	-	-	-	-	-	-	-		-	2.08 %	-
	Malaria	5.06 %	8.80 %	11.54 %	-	4.51 %	4.49 %	19.64 %	17.41 %		29.17 %	14.43 %
	TB	-	-	-	-	-	4 %	-	-	-		6 %
	HIV and AIDS	-	-	-	-	-	-	-	-	-	3 %	

*Note. IHD* ischaemic heart disease, *HTN* hypertension, *DM* diabetes mellitus, *COPD* chronic obstructive pulmonary disease, *TB* tuberculosis, *HIV and AIDS* human immunodeficiency virus/acquired immune deficiency syndrome

The individual prevalences of diseases are organized circularly along the periphery of the wheel. Ribbons connecting segments refer to a comorbidity between the two diseases represented. The relative width of lines is proportional to comorbidity rates. Each disease is assigned a specific colour; comorbidities have the same colour as the origin and the width indicates its relative prevalence in the study population.

Malaria cases represent the largest portion of the multimorbidity wheel’s outer ring (Fig. 1), with 5310 recorded cases, followed by diabetes with 1400 cases. From a broader disease perspective, NCDs represented 38 % (*n* = 3401) of all categorized cases, with the majority of NCD cases being type 2 diabetes (*n* = 1440) and hypertension (*n* = 1398).

Malaria also presented most frequently with at least one NCD comorbidity. Among malaria cases, 19.6 also had COPD, followed by 17.4 with asthma, 11.5 with stroke, 5.1 with IHD, 4.5 with type 2 diabetes, and 4.5 % with any type of cancer. TB, meanwhile, overlapped with hypertension (4.2), cancers (4.0), and asthma (2.1 %). HIV and AIDS did not appear comorbidly with any of the measured NCDs, though a substantial number of people living with HIV and AIDS also presented with malaria (14.4) and TB (6.0 %).

## Discussion

Using novel visualization techniques, this study presents the complex weave of NCD-ID interconnectedness at work among employees of a major mining operation in a remote, generally under-resourced, area of Indonesia. The findings have important implications for health system reform both at the clinical and at the broader, public health level.

### Clinical pathways and implications

A gamut of overlapping agents, pathways, outcomes, and co-factors characterizes the recognized correlations and complex causal links between NCD and ID burdens [14]. The basis of these overlapping burdens is both biological and social, involving augmented risk of disease development and/or exacerbated disease severity, with bidirectional interrelationships in some cases. For example, diabetes and tobacco smoking increase susceptibility to TB [15], and between 15–20 % of cancers have infectious origins [16]. Meanwhile, the risk factors associated with poverty, unhealthy lifestyles, tobacco use, and alcohol abuse are common to NCDs and IDs alike [17–19].

The association between malaria infection and risk of more severe TB outcomes is known [20–22]. In this workforce study sample, cancers were connected to both malaria and TB cases. Considering that the study was conducted in a high-burden TB and malaria area (with the highest comorbidity rate recorded between these two IDs), this may be incidental, similar with the variety of NCDs also observed in combination with malaria. However, a potential causal connection is supported by an extensive field of oncological-ID associations [23]. At the molecular level, infectious agents may be significant initiators of mutations and the dysregulated cellular proliferation that follows [24].

For cardiovascular disease (CVD), recognition of the link with IDs, as observed between malaria and stroke, IHD, and hypertension, and between TB and hypertension in this study, dates back to the late 1800s, and more recently in research associating CVD primarily with behavioural and genetic risk factors [25]. There is an increased risk of CVD in the presence of infectious agents such as *Helicobacter pylori*, cytomegalovirus, periodontal bacteria, and *Chlamydophila pneumonia* [26]. Antibiotic treatments for the prevention of cardiovascular events and modified lipid metabolism following viral infection [27] are two other associations linking both disease groups.

The NCD-ID connection may also be mutually reinforcing, as reflected in the asthma-TB overlap highlighted in this study. Chronic lung diseases have been identified in various settings as a risk factor for both TB development [28, 29] and TB recurrence [30]. Their frequent comorbidity is likely because the two diseases share a common set of risk factors; prolonged exposure to cigarette smoke and air pollution are recognized risk factors for development and exacerbation of asthma [31, 32] and TB [33].

### Public health implications

The compounding effects of multiple NCDs and IDs underscored in this mining community necessitates a re-thinking of the current model of policy planning and healthcare delivery to better combat these two colliding maelstroms. Particularly in resource-limited settings [12], disease-specific approaches do not represent the most efficient response [9, 11]. Recognizing that NCDs and IDs share linked origins, the health systems to cope with cancer and diabetes, for example, share many similarities with those needed to handle common IDs. More traditional approaches to global health need to be reassessed, with greater emphasis on multidisciplinary collaboration and site-specific integrated strategies [34].

Indonesia is currently proposing major health system reforms, which presents a unique opportunity to combine both NCD and ID challenges with integrated novel approaches [9]. Indonesia aims to provide equitable and essential-level healthcare country-wide via a universal healthcare scheme, which is a significant departure from the existing health system delivery model. The increased cost associated with expanding healthcare will likely impact or preclude other major capital infrastructural investments needed in Papua, necessitating utilization of the existing resources to deliver coverage for escalating NCDs. For example, the integration of diabetes monitoring into existing point-of-care electronic medical record systems for directly observed TB therapy in Malawi [35], or the establishment of NCD clinics that integrate HIV and AIDS care with the management of diabetes and hypertension in Cambodia [36] demonstrate the possibility of merging service delivery for both disease groups without the need to significantly scale up existing infrastructure. Similarly, in China, India, and other countries with high burdens of both disease groupings, large-scale programs for dual screening and management are now being rolled out [37].

A major challenge of ID control is the improvement of epidemiological surveillance and prevention, case detection and management, and increased efforts towards the elimination/eradication of diseases. Meanwhile, the reduction of metabolic and behavioural risk factors and unhealthful environmental exposures are among the outstanding challenges facing NCD control. Therefore, NCD and ID prevention and control efforts need to be integrated in the most cost-effective manner possible, focusing efforts on conditions with the highest burden, especially those that could impact a wider array of aliments.

Within the regulatory space of occupation health, Indonesia has made grate strides to adhere with international norms and best practices as is the case of HIV and AIDS [38]. However the Ministry of Manpower, the regulatory body in charge of overseeing policy development and implementation of occupation health standards has yet to implement regulations targeting the number one cost driver within workforce populations, NCDs [8] Adopting regulatory measures to ensure both private and public entities allocate resources to NCD prevention not only falls in line with current international recommendations but will increase the nation’s ability to provide the best population health outcomes for its people.

### Primary healthcare system strengthening

A focus on NCDs can contribute substantially to strengthening the health system [39]. To address NCDs, a set of functioning fundamental services for primary prevention, screening, early detection, diagnosis, and treatment, alongside systems for referral and follow-up are needed, supported by adequate training of staff. Ultimately, the double burden of disease requires integrated control approaches that should begin in the primary healthcare sector, with a focus on health promotion and disease prevention strategies [40]. An optimal public health response would target all common causes of disease, with multiplied returns through investments and interventions that address the causes underlying both IDs and NCDs (e.g., food and nutrition policy, housing policy, employment, and provision of healthcare [10]).

## Conclusions

Findings among mine workers in Papua, Indonesia highlight the interrelationship between NCDs and IDs, suggesting areas of potential synergy and cooperation in terms of reducing comorbidity, risk, causation, and provision of more comprehensive healthcare to a larger population. The novel visualization of overlapping areas in the form of the multimorbidity wheel draws attention to the intersections of various disease burdens illustrating relative commonalities, thus showing areas where further exploration toward integration might be more meaningful and successful. Because of the strong links between NCDs and IDs, it is prudent that the two be addressed in a complementary fashion by health financing and delivery systems.

The dearth of successful, large-scale, integrated NCD-ID programs in LMICs challenges the health community to rethink the future direction of global health. Moving forward, public health research should strive to produce pragmatic and site-appropriate solutions that integrate NCD prevention programs into existing ID structures via patient-centred, primary healthcare delivery models [36]. By convening to align future strategies at this crossroads, stakeholders from each side will benefit from the other’s experiences and lessons learned in healthcare delivery and research. Rather than NCDs and IDs being positioned as disparate factions competing for limited resources, the time has come for concerted moves towards a non-competitive synergy to tackle both scourges head-on.

## Abbreviations

COPD: Chronic obstructive pulmonary disease
COPPER: Cardiovascular Outcomes in a Papuan Population and Estimation of Risk study
CVD: Cardiovascular disease
DOTS: Directly observed treatment, short-course
HIV and AIDS: Human immunodeficiency virus/acquired immune deficiency syndrome
ICD-10: International Classification of Diseases, 10th Revision
ID: Infectious disease
IHD: Ischaemic heart disease
LMICs: Low- and middle-income countries
NCD: Non-communicable disease
TB: Tuberculosis.
